# Of Great Apes and Magpies: Initiations into Animal Behaviour

**DOI:** 10.3390/ani10122369

**Published:** 2020-12-10

**Authors:** Gisela Kaplan

**Affiliations:** School of Science & Technology, University of New England, Armidale, NSW 2351, Australia; gkaplan@une.edu.au

**Keywords:** animal initiated contacts, ethological methods, perspective taking, orang-utans, juvenile magpie, empathy, unselfing, contact zone, cognitive package

## Abstract

**Simple Summary:**

Animal encounters have been favourite subjects for a long time and it would scarcely be novel to report such stories for their own sake, even though the ones told here are dramatic enough to stand on their own. The questions addressed in this paper are twofold. The first question is: What influence may particular and dramatic animal encounters have on the human observer and how dependent is such a response on previously held attitudes? This paper provides three cases studies of extraordinary moments that changed the lives of the human participants and turned them into advocates of the species they had encountered. The next question asked is how we can be respectful of animals without anthropomorphising them and study them in ways that help us understand their abilities and their needs rather than impose questions that mean much to the human researcher but could be irrelevant to the species? The examples given here compare and contrast species that are especially close to us (great apes) with studies of those that are distant from us in their evolution (birds) and show how different attitudes change the questions that can be asked by scientists, demonstrably leading to new and even stunning results.

**Abstract:**

This paper presents three case studies of exceptional human encounters with animals. These particular examples were selected because they enabled analysis of the underlying reasons that led the human participants to respond in new ways to their animal counterparts. The question asked here is whether sudden insights into the needs and abilities of an animal arises purely from an anthropocentric position as empathy because of genetic closeness (e.g., chimpanzees) or is something else and whether new insights can be applied to other phylogenetic orders not close to us, e.g., birds, and change research questions and implicit prejudices and stereotypes. Particularly in avian species, phylogenetically distant from humans, the prejudices (anthroprocentric position) and the belief in human uniqueness (human exceptionalism) might be greater than in the reactions to primates. Interestingly, in studies of great apes, contradictory opinions and controversies about cognitive abilities, especially when compared with humans, tend to be pronounced. Species appropriateness in test designs are desirable present and future goals but here it is suggested how different experiences can also lead to different questions that explode the myth of human uniqueness and then arrive at entirely different and new results in cognitive and affective abilities of the species under investigation.

## 1. Introduction

Claims of human exceptionalism are not new and, over the centuries, have been expressed in different forms and arguments, be these focussed on the soul, reason, consciousness, morality or language. As Pickering said, we have always found something humans have that “the rest of the world doesn’t, and that something has always been the object and the raison d’étre of the human sciences” [1].

Human uniqueness is reconfirmed everywhere, even in papers, books and contexts where such statements seem superfluous. They are stand-alone claims to celebrate and confirm human superiority. The word uniqueness, an argument usually not mentioned, is actually only justified in relation to the stunning extinction record of *all* human ancestors, possibly as many as 13 species of *Homo* (and others are still being discovered), such as *Homo habilis, H. erectus*, *H.ergaster*, and *H. antecessor*—the latter the common ancestor of Neanderthals (*H. neanderthalensis)* and modern humans, *H. sapiens*. *Homo sapiens* is the last and only survivor of that long line of hominins and more predecessors have been found recently, including the Flores people (*H. floresiensis*) that went extinct possibly 50,000 years ago [2]. The link between the current species and previous ones, called “archaic humans” remains hotly debated but more scientists are coming to the view that there are admixtures between modern humans and archaic populations which introduced additional loci in the human genome [3]. Perhaps all this bravado and the public pronouncements of human uniqueness are also expressions of a deep-seated dread that modern humans are on their own, out on an evolutionary limb and there are few species in that precarious position today.

Beyond these biological and anthropological observations, the claim of “uniqueness” is actually a tautology because all living things are “unique” if unique means that they can only breed within their species. If the claim of uniqueness extends to social, cognitive, and other attributes, this claim would again be false because we keep finding antecedents of biological, behavioural, and cognitive ingredients in many vertebrates and even invertebrates [4].

In human thinking, the position of primates, and especially of great apes, may at times be particularly uncomfortable because of their proven relatedness to humans. On one hand, primates have been studied extensively and over a very long time, precisely because they are close to humans and a good deal can be learned from studying the extant primates in order to establish which traits persisted. By the same token, the “nearness” of great apes to our own human biology and cognitive abilities, has continued to create unease and discomfort leading to extraordinary efforts to disprove their nearness. In 2010, an edited volume called *Mind the Gap*, invited comparative articles (comparisons with primates) on core aspects of behavioural and cognitive traits “that make humans such unusual animals” [5]. The writers contributing to this volume, with some exceptions, argued throughout, particularly in a section on cognition and communication, that chimpanzees cannot compare to humans in any meaningful sense of the word, including communication. The ambivalence expressed in this volume occurred long after all the experiments of learning American sign language with bonobos, chimpanzees, gorillas, and orang-utans had been completed and published [6].

From a point of view of evolution and biology, “uniqueness” ultimately makes no sense whatsoever. Humans, as mentioned before, were not the first species to have a brain, a perceptual apparatus, or means for locomotion. Indeed, the basic building blocks of life, apart from its chemistry, have now been found to include a basic “cognitive package” common to all vertebrates and humans, as has been so effectively shown in research using chicks by Giorgio Vallortigara and colleagues [7,8]. This cognitive package was foregrounded by what Elizabeth Spelke (2000) called core knowledge, also evident in infants [9].

In 2018, Finlay [10] published an interesting paper called “Human exceptionalism, our ordinary cortex and our research futures” which is rightly critical of the emphasis on the cortex as the seat of all that is worth knowing about the brain. She said that “our understanding of the computational complexity of the entire brain has been systematically underappreciated as a result of our mistaken fixation on the neocortex”. In the very same article on the human brain it also speaks of such (allegedly) unique human traits, such as “behavioural complexity, or intelligence” [10]. One could take umbrage at these statements because countless papers have demonstrated that these supposedly “unique traits” are not uniquely human at all but are spread across a wide range of vertebrates including birds. For these traits in animals there is substantial scientific evidence apart from the primate line, be this in cetaceans [11], corvids [12], parrots [13], other songbirds [14], dogs [15], elephants [16], and many more.

Admittedly, it is also easy to over-interpret and paint elaborate pictures of minds in animals that, as Cimatti and Vallortigara rightly said [17], could be explained in much simpler terms. Parsimony is still a golden standard in science. However, one suspects that some of the most earnest rejections of cognitive qualities are also derived from researchers with the most anthropocentric views, driven by a deep-seated need to maintain the status of human superiority at any cost even when robust evidence says otherwise.

This paper will deal with exceptional encounters with great apes and with a juvenile bird, an Australian magpie (*Gymnorhina tibicen*) in very different circumstances but all having a deep and lasting effect on the human participant. However, extraordinary events, no matter how touching at the time, do not necessarily lead to changes of minds and break the cycle of the conviction of human exceptionalism/superiority. I am claiming that empathy is the kind of response that may be of short-term value but ultimately not lead to a shift in thinking about animals. Empathy can be patronising, merely reconfirming the human observer’s superior status. It can be expressed as pity and sorrow without making the slightest dint in human perception of “naturally” having to exercise control. Instead, a prosocial view of all things around us may make us think of animals as communities that we may be privileged to share and, if we do respect animals and the natural environments on those grounds, we may be granted some insights into how their lives have adapted to niches in the world that may have taken millions of years to perfect. It is a process, Zhang and colleagues called “unselfing” [18]. They suggested that people in the natural environment might become more open to socially friendly contacts (being prosocial). “Unselfing” is quite an innovative way to describe a possible alternative meaning, but I like to use it not quite in the way Zhang et al. used it. The authors [18] understood it as an improvement in prosociality among humans as a consequence of being in nature. “Unselfing” could also describe a process of treating other living things, not humans, prosocially and thus improving our understanding of the species that we study. Perhaps this is what Williams et al. [19] had in mind when they advocated a mindful approach to animals. The ultimate test question one may ask: If all humans disappeared tomorrow, would the earth recover and flourish? It is a fact that, if all the non-human animals disappeared, humans would eventually go extinct. One’s approach to science, while embedded in very long traditions of excellent methods and techniques, is ultimately also informed by our own culture and by our prejudices but some (most?) of these could also be unlearned [20].

## 2. Material

The material consists of three case studies presented as narratives.

### 2.1. Case 1

The International Primatological Society holds conferences every two years alternating between habitat (primate) countries and non-habitat countries. In 1996, its XVIth Congress was held from 11–16 August in Wisconsin, Madison. Its program consisted of the typical range of keynote addresses, regular papers and posters [20]. As usual, it also offered some roundtable discussions. 

Offerings of roundtable discussions included an advertised theme called was “Breaking the Silence: Enhancing the use of personal experience in primatology” and it asked participants to think about four different topics within this theme:

(1) In research: How do we move from personal experience to useful hypotheses?
(2) Care-taking: When does intuition about non-human primate well-being supersede protocol?
(3) Education: How does empathy advance the process of learning about other primates? and
(4) Conservation: What impact does personal experience have on the ethos and effectiveness of conservation?

The roundtable was allocated two hours but nobody left after two hours and discussions went on well into the night. Several speakers were scheduled to give short talks to help structure the time. As the talks unfolded it became clear that the people who spoke, often had had profound experiences with a primate or primates that affected their entire lives and even changed careers and fates.

It was, in short, an intense period of emotional outpourings from academics used to the rigours of research and distancing themselves emotionally from the very animals they were studying. Here was one of the rare opportunities to turn attention to the motivations, emotions and thoughts of the individuals who had chosen to study primates rather than on the results of their research on non-human primates, a forum for voicing and possibly unlearning former biases [21].

One of the speakers who stood out was James Mahoney (1940–2017), called posthumously “the Oskar Schindler of laboratory primates” [22]. A softly-spoken Irish veterinarian who had worked on cattle in Scotland and had moved to New York for postgraduate work, recounted his time of employment with the Laboratory for Experimental Medicine and Surgery in Primates (LEMSIP), a private facility of the University of New York in Sterling Forest, New York. Chimpanzees and other nonhuman primates were subjected to intensive biomedical research in areas including reproduction, blood transfusions, and hepatitis B, working also on a cure for AIDS and using live chimpanzees to test AIDS and chemical compounds in combatting the disease. Mahoney joined LEMSIP in 1975 and participated in the AIDS research and actively tested AIDS on chimpanzees from 1988 onwards.

The chimpanzees were housed in what most onlookers would perhaps nowadays consider appalling conditions—small steel cages with bars all around, and the floors were also steel bars. There was not even a platform where the chimpanzees could rest: The cages were lifted off the ground and hung like bird cages in the laboratory. The chimps had no access to any outside enclosure. Females were forced to reproduce each year and their offspring taken away within a year to become part of the laboratory’s biochemical research activities. Mahoney gave medications and injections to the chimpanzees for over a decade and, given that there were only six suspended cages in his laboratory, the chimpanzees knew him well.

The main point of his talk consisted of describing in detail his return to work after a bout of illness and absence from the laboratory. And then it happened in one fleeting moment. On returning to his laboratory Mahoney tried to settle back into his routine and that included checking on “his” chimpanzees. As he approached, one chimpanzee who had probably suffered the longest and over years, extended her arm out of the cage and used her hand to briefly but gently stroke Mahoney’s arm. At that point in his talk, Mahoney was visibly moved choking back tears and found it difficult to talk on.

The gesture coincided with a time when a deal had been made with another laboratory to transfer the chimps to them, contracts had been signed and the chimpanzees sold were to be transported to that facility shortly (not without major public outrage and court cases instigated by the other laboratory). Literally instantaneously, after being stroked by the chimpanzee, Mahoney had one clear thought in his mind and that was that he somehow had to save the primates. Without hesitation Mahoney organised trucks, crates, and an army of volunteers starting the slow process of stealing chimpanzees from the research facility under cover of darkness. The research facility LEMPSIP at that time held about 300 chimpanzees and nearly 300 monkeys [23]. He managed to get over 100 chimpanzees out in the first instance and by 2002 the remainder of the primates had been shifted to sanctuaries.

He also held a position at a local university with ties to LEMSIP but was never fired nor was the matter raised by the university despite the loss of hundreds of thousands of dollars that these primates had cost the university and the institute. The media were heavily involved and the matter was publicly debated everywhere. Mahoney became a hero to some and an animal right’s activist (of the worst kind in some people’s views) worthy of sustained attacks to others. He had saved the chimpanzees and they lived out their lives in the company of other chimpanzees in sanctuaries and facilities especially designed as comfortable “chimp retirement homes” with outdoor spaces and activities.

### 2.2. Case 2a: Encountering Abbie

The second and third cases of extraordinary and career-determining moments, are my own. These are equally within hindsight of about 25 years, as the above. Before they occurred and changed my life, my first academic career was that of a social scientist, researching and writing about prejudice, racism, sexism, and biological determinism. These pursuits had produced books on local and international women’s movements and women’s status and on the politics of survival (first PhD thesis title). Within six years of completing this first Ph.D., I was appointed professor of social science and, by that time, had a strong record of published papers, also seven books including an edited book on Hannah Arendt. While the appointment was made on the strengths of publication in social science it was made also on equal training in sociology and psychology. The research focus of this first career was thus far removed from the second academic career in ethology/animal behaviour. Working in the field of Animal Behaviour for me largely meant undertaking field research on primates and birds but in the last 15 years almost exclusively concerning birds. These research interests also determined our PhD programs and they have been fertile years which have produced some 16 books and a large number of research papers.

The events related below not only belong to the second career but were the very reason for it. This second academic career began in a somewhat unusual way and started with a flight to a conference in Europe. Coming from Australia we decided on a stopover as Malaysia Airlines had advertised free side-trips to Kota Kinabalu for long-distance travellers to open up Sabah for tourism [24]. We promptly arrived in Kota-Kinabalu. Because of work-constraints, we only had three days and chose to fly on to Sepilok, the orang-utan rehabilitation centre situated outside Sandakan.

There were quite a number of books available on Borneo, and that has historical reasons. For 100 years (1867–1967), the Malaysian provinces of Sabah and Sarawak were under British colonial rule and called British North Borneo.

I was very keen to visit Borneo and this eagerness stemmed not from travel brochures which were not really yet available in the 1980s and early 1990s, or some overwhelming desire to go off the beaten track. Having been raised in large cities and working in large cities aroused little interest in jungles. The interest came from books and short stories I had read as a child. One particular one was the character of King Louie (modelled on an orang-utan) in an early film of Rudyard Kipling’s *Jungle Book* although taking license of putting orang-utans into an Indian jungle. He played a role, but also, and perversely, a frightening children’s story called “Earwig” that was set in Borneo.

When we landed in Sandakan, we took the next local bus out to Sepilok. A local bus then was something to behold, both for its age and the smoke bellowing from the exhaust and the seats, torn and battered. The seats were so small that we each had to occupy two seats to fit and then sit sideways because our upper legs were too long for the space provided. At 5.6 feet I am neither particularly tall or broad—the locals were just a good deal smaller and daintier who, in good humour, in later years dubbed me “Rambo” putting on a wide smile. The bus rattled us through small villages and occasionally stopped at small plantations until we arrived an hour or so later at the Orang-utan Rehabilitation Centre. A few locals wearing uniforms like our western wildlife rangers, a veterinarian and a few other officials were there, a few houses and cages for injured orang-utans were barely visible behind bars in the distance but otherwise the place was deserted. We asked what we can do. One nice ranger just motioned us on and said that we can go. Hence we started our walk into this forest, soon more looking like the real tropical rainforest and the “jungle” I had always wanted to see and we were alone, going deeper and deeper.

The heat was forbidding—only about 32 °C but it felt a good deal hotter because of the high humidity and the absence of even the slightest breeze. There were some orang-utans walking across the forest path in the distance and disappearing until we came to a clearing where several juveniles were sitting on the floor or playing with each other. This clearing, in a sense, came to symbolise what Donna Haraway called a “contact zone”, a multi-species meeting that was entirely unforced, unstructured, and significant [25], adopted from Pratt’s influential book *Imperial Eyes* [26]. We stopped at the edge of that clearing, just watching and keeping our distance. We learned later that some of the orang-utans were orphans, usually a fate inflicted on them by humans involved in the illegal trade of orang-utans, supplying a surprising number of zoos worldwide.

Eventually, the orphans started coming over to us—we were crouching on the floor initially but a cramp forced me to stand up. As I did so, one of the juveniles headed straight for me and crawled up holding on to my neck and making herself comfortable on my hip. This, as I recalled later, was the moment that changed my life. At the time, I was so perplexed and without any substantial experience of animals, had not ever even been to the countryside or experienced cows or pigs. I had no single learned strategy on hand on how to deal with this. The orang-utan, about 4 years old, was quite hefty in weight (perhaps about the weight of a 2-year-old, 8–10 kg) but I just let it happen. There seemed to be nothing aggressive about her and, reassuringly, she purred with a low grunting sound, literally like a cat, and first inspected my face with her hands that were uncannily similar to a human hand and were soft and warm. She then turned her head and rested it on my shoulder. She had a lovely scent—a beautiful lemon-scented/orange type of smell emanating from her body. Her skin was soft and white and one comparative look at her arm and mine showed the same soft clean and unmarked skin—hers was covered in long but sparse red hair—as I found out through the afternoon, the hair entangles mosquitoes and prevents orang-utans from being bitten. I do not really remember any details of what we actually did—it was a dreamlike, still afternoon despite the heat and the weight of the girl, later identified as Abbie, who added an extra blanket of heat around my body. Her weight was beginning to be difficult to carry, even though Abbi helped by having wrapped her legs around my hips in such a way that the main weight was now carried by my hips, not my arms.

Such encounters would not happen today as mass tourism became the norm over the years. The transition from a wild place to a tourist attraction eventually reestablished some of the asymmetry of power between animals and humans, between former colonials and western imperialisms [26,27], because tourists and even volunteers, paying high fees for the privilege to be with orang-utans [28] expected and even insisted on interacting with orang-utans. By this insistence and monetary contract, rehabilitant orang-utans were transformed into attractive products for the travel industry. Incidents, including acts of aggression, violence, and injuries by human visitors and orang-utans alike [28] later made it imperative that contacts had to be forbidden by the administration for health and safety reasons. But there were no tourists at that time at all. We were entirely alone in the forest.

It all ended abruptly once we noticed with a start that we had to get out of the forest and back to the bus stop at once since the last local bus left the rehabilitation station at 5 p.m.—after that it was also getting dark and it was far too far to walk back to town. As we got close to the buildings I asked that same young man to please take Abbie and he did and we walked away. I dared not look back but heard the loud and continued screaming/crying from her that chilled me to the bone. In walking away I knew I had to come back and see her again.

And we did come back many times and Abbie “visited” us every time/every year when we went to Borneo. The last time we were in the forest, Abbie, aged 9 by then, was nowhere to be seen and none of my calls were answered.

**Figure 1:**
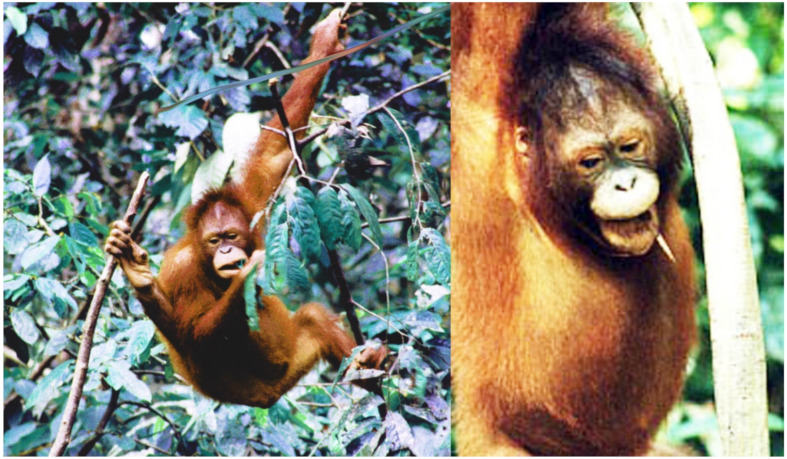
(**Left**): The nine-year-old Abbie is showing off, swinging in front of us- a special “performance”–amongst vines that are difficult to negotiate. Note her foot, more like a hand, which can be used to push herself in the opposite direction or to pull herself forward to the next vine. (**Right**): Abbie dangling, showing her playface (upper lip tightly drawn over teeth) (Credits: G. Kaplan).

A day or so before we had leave I tried calling her for the last time and then saw an adult looking orang-utan amble towards me, slowly and deliberately. When orang-utans reach this sub-adult stage, they can change quite dramatically in physical appearance. Uncertain about the orang-utan’s identity, I decided to sit down on the forest floor and wait, largely eyes averted. She came straight towards me—even though other people were around but not close by—and with a jump of my heart, was overjoyed that she was sitting down opposite me. She sat and looked this way and that and then slowly took both my hands and held them, then she dropped one hand, turned my hand around and ran her fingers over the palm of my hand, slowly and deliberately with her index finger, studying my hand intensely. Then she gave me back my hand, took hers back and we just spent a good deal of time sitting there. A little later, details also recounted elsewhere [29], her mood changed. She suddenly got up and disappeared but returned quickly, this time with her friend Alice, and then the two gave a performance of frolicking and play. Abbi wore her play face (Figure 1) and eventually the two slowly disappeared together in the forest. My audience was over and it was the last time I saw her.

### 2.3. Case 2b: Simbo and Danger

**Figure 2:**
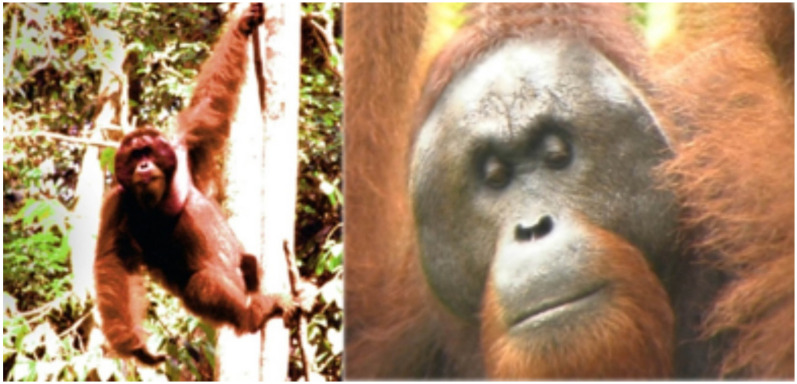
(**Left**): Adult male orang-utan (Simbo), an imposing figure, some 80 kg in weight, nimble, and very strong (note the strong, long arms). This was the moment when he appeared at the other end of the clearing, some 50 m away. (**Right**): The head of an orang-utan male is considerable larger than a human head, exaggerated by the cheek pads (flanges) (Photocredit author).

Many of the orang-utans that had been there at the time of our first visit had grown up and been released in the Danum Valley but some were able to stay, including a strong adult male who had been treated once for a broken limb, but that was over 20 years ago. He now ruled over this vast territory and occasionally came within a few km of the Centre, sometimes just to partake in the food that was offered to rehabilitating orang-utans high up in a tree on specially built feeding tables. At times, when fruiting trees were sparse, Simbo arrived at one of these feeding stations, usually one of the most distant ones from the rehabilitation buildings and without people, announcing his imminent arrival with a spine-chilling so-called “long call”, somewhere between the sound of a male moose or deer in rut, the trumpet of an elephant with the threatening undertone of a dark roar of a lion. It was so loud, despite the deafening continuous sound of crickets and other insects in the forest, that his bellowing could be heard over a kilometre away. An adult male cuts an imposing figure and, with large side-flanges on either side of his cheeks looks even larger than he actually is (Figure 2). Simbo, while he had been in contact with humans during his fracture treatment, was considered a wild orang-utan whose only difference from completely wild orang-utan males was that he had lost his fear of humans and was now considered dangerous.

That particular morning, my team partner Lesley Rogers and I had separated in the forest so that we could maximise observations, scoring and filming their behaviour, across a larger number of orang-utans. It was not a time for provisioning the orang-utans, happening daily when one could see many of them congregate at feeding tables attached to trees at some height. Orang-utans knew the routines and, not expecting any handouts, had spread out over great distances in the forest. After years of visiting the forest we were now familiar with the fifty-or-so orang-utans in that region and could distinguish them individually. We had gone a good way into the forest when I suddenly realised that we were no longer in visual contact with each other and it was not clear in which direction Lesley had gone. There were no paths to follow, just the natural forest floor. Looking around I found myself entirely on my own. That is when Simbo called. Any orang-utans in visual distance had suddenly disappeared and, in the distance, the unmistakable shape of an adult male orang-utan appeared.

Since visits of the adult male were rare in that part of the forest, I decided to stay and set up the camcorder on a tripod and started filming him in the distance. Without warning, his measured movements changed pace. He had spotted me and swung very rapidly from tree to tree directly towards me. It is astonishing how fast orang-utans can move—faster than any running could have carried me. Within minutes he had reached me, stopping short a mere two metres away and about two metres off the ground, clasping a branch and half-sitting on a tree branch. He was uncomfortably close, almost face to face. We had learned some orang-utan etiquettes in the meantime: Never look directly at an orang-utan. Direct stares, even in some human cultures, are considered “rude” or worse. In orang-utan and quite a few primate societies it can signal daring, insubordination, or even outright aggression. Keeping very still, with eyes averted and downwards I watched him on the video screen and waited, barely breathing. The swipe of his arm alone could break my neck and kill. Orang-utan males have about seven times human strength. Indeed, he could lift me with one arm, as actually happened many years later in a confrontation between a tourist and another male (Raja) who stripped a woman off her clothes and dragged her up a tree. Orang-utans also have impressive canines which are used effectively to open a fruit called Durian but they can also be used for biting.

Simbo sat still as well but despite his inactivity I knew he was inspecting me. Rerunning the tape later, it became clear that he had indeed furtively glanced at me every few seconds even when his head turned as if he were looking somewhere else. As we learned from analysing the data of eye gazes of many orang-utans later, Simbo’s behaviour was typical, snatching actual looking time in brief glances but making it appear as if not looking at all [30]. The silence between us, despite the deafening noise of the forest insects and suspended waiting time seemed endless. Standing motionlessly in the hot sun, perspiring profusely, with sweat from brows finding its way into my eyes and stinging, dripping from ear lobes, even from the tip of the nose, legs stiff with tension and beginning to see spots permitted some reflection. When the tables were turned, as in these moments, a realisation of being defenceless, exposed, and humbled struck hard. Simbo finally decided to leave, in an unhurried and even hesitant fashion. In this silent round of wills, he certainly came out as the uncompromised winner, but I had survived unharmed. I waited and continued to stand totally still until the distance between us seemed safe enough to move; however, my legs were shaking so badly, they knocked over the tripod with the camcorder. Perhaps this experience ranked as one of my most heart-stopping encounters in the wild.

Our days in Borneo’s rainforest came to an abrupt end. We had worked with orang-utans for many years reflected in books and papers, published in Australia and overseas [30,31] endorsed by the Orang-Utan Foundation. Political events well out of our control were to blame. The Australian and Malaysian prime ministers, for reasons of their own, had personalised their differences expressed in tit-for-tat public diplomatic spats that often did not even make the news. One of the consequences was that the Malaysian government cancelled all research permits and activities for Australian researchers. We may have been spared seeing the dramatic deforestation of Borneo and the rapid decline of orang-utan numbers since our presence there but the sudden and involuntary severing of ties with the forest and its orang-utans has remained a lasting and even melancholic loss.

Professionally, the loss of the orang-utan research barely made a dint. At that time I was full professor in social science and headed a large school by that name, was an editor of one of the chief journals in Australia and served on most major committees in government and, in the academic discipline, had written several books, including orang-utan books and papers, derived from studying very specific aspects of rehabilitant orang-utan behaviour [32], and one should have thought that this was satisfying enough and could have served me well to the end of my working days. The movement from human research to studying great apes was condoned. After all, many primatologists had built their careers on the discipline of psychology. Hence, however profound the experiences were by themselves, they did not change my daily life.

Yet these experiences in Borneo had left other indelible marks, making me wish to do something for native wildlife at home. I joined a volunteer organisation for the rehabilitation of wildlife and specialised in hand-raising and rehabilitating native birds in my spare time, something that I have now done for over 25 years.

This step was again a portal into a world I knew little about. There is nothing more satisfying than helping an animal back on its feet that would have been doomed otherwise and reintroducing it back into its community. It was a privilege to get to know so many native birds I had never seen before. I was acting in loco parentis, raising individual nestlings belonging to many species entirely unafraid of me and showing the range of behaviours they might show to their parents. The organisation is governed by a strict set of rules which also includes that any bird under a person’s care must not be used as a pet, must not stay longer with the carer than absolutely necessary, must be releasable, and cannot be sold, loaned, or used in any way other than for the purpose of safe release back into the wild and that also, of course, included that any holding of a bird for the purpose of research was and is strictly against the rules. However, putting the caring role together with a very active interest in animal behaviour made it inevitable that one noticed behaviour.

Clearly, the orang-utan years had provided an entry point to animals and nature in general or indeed to a multi-species “contact zone” [25,27] that, once entered, cannot ever be left behind or unimagined. No doubt, these experiences may have altered my thinking and in ways that had not been consciously processed. The orang-utans have never faded from view but since 2002 I have never seen another orang-utan in the wild again.

### 2.4. Case 3

A decisive experience with a bird may probably not sound as dramatic and might lack the exotic flair and adventure of a Bornean jungle story and of orang-utans. Yet it was this bird, an Australian magpie juvenile *Gymnorhina tibicen*, that was directly responsible for setting into motion an entirely new path in life, including my complete career change.

**Figure 3:**
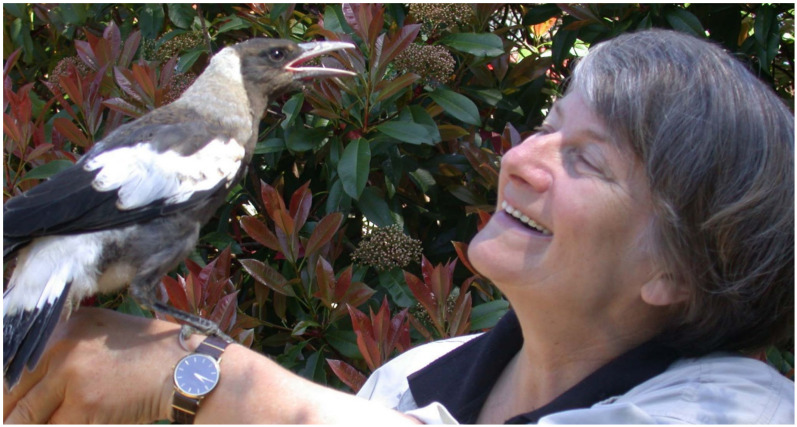
Author interacting with a juvenile magpie many years later but looking very similar to the bird that had such profound influence (Credit: L.J.Rogers).

This single juvenile magpie had been hand-raised and released into our garden. As it turned out, the bird later formed a family in a near neighbouring plot and stayed there for the next ten years raising its own offspring with its wild partner, that achieved this life changing transformation in my life (see Figure 3). It catapulted me out of the chair, back to studying and undertaking a second PhD, back to a very junior position but now in science. And all because of a bird, the way it presented itself to me as if I were a play-mate and a personal confidante. When I moved into the garden the bird shot over to me and immediately tackled my sneakers or, rather, the shoe laces making me hobble through the garden. The bird did somersaults in front of me, hung by one leg upside down from the near apple tree but only if I was close-by. Its need for playing was extensive and we pulled grasses from beak to hand as a tug-of war competition, played hide and seek, competed for objects, and explored the property together including finding juicy morsels. It was a lovely relationship.

But it became more than that. One day I heard a clear voice from outside the living room, annunciating: “I have got dinner for you”. Since I am usually not the cook but the recipient of food, these words were not copied from me but, most likely from my partner. They had the same intonation and urgency, usually required to get me roused from my desk and the computer. Looking everywhere for the source of this authoritative statement, the mimicry was perchance repeated when casting my eyes over the garden. I could clearly see that it was the magpie performing this vocal mimicry.

This was entirely unexpected and stunning. It made me set up a tape recording and a microphone outside at once, recording everything the magpie uttered. I began to miss luncheon appointments and skipped social engagements because of the compulsion to record the magpie. Whatever for? one might ask. There was no single good reason but then, this was not unlike a process of madly falling in love, so powerful that the trajectory of actions that followed made absolutely no sense at all from any work or career perspective. Indeed, it seemed rather suicidal in terms of career and workplace.

It was also clear that if “studying” magpies, or any birds for that matter, were to have any value into the future, and were not just to remain a private little hobby, my academic background was simply not sufficient. I have no idea to this day why I decided to call the School of Veterinary Science in Brisbane which I knew had a particular strength in animal behaviour, and asked to become a student again and there it was: Without ado, Professor Kaplan was accepted as a Ph.D./graduate student, having to do extra courses because of an obvious lack of knowledge in biology. The thesis completed besides almost full-time work was about the vocal development and behaviour of magpies, a 100,000 word tome and the result of four-and-a-half years of recordings, measuring, field work, and study, by then not just depending on the few magpies in my garden but having set up five major research sites in suitable distance from each other and spending substantial amounts of time in the field, recording thousands of hours of vocalisations and noting down which behaviour preceded and followed any utterances, then spending nights at the computer transcribing the tapes and transforming key ones into sonograms. If there was a magpie anywhere, even if during a meeting, I took notes not of the meeting but of the magpie. Thirteen nest sites were identified for study over several years and visited almost daily. Wherever possible, microphones were affixed near the nest to record the nestlings’ vocal utterances throughout the 4-week nesting period and I even filmed nesting activities from a 4-metre guy-roped ladder swaying precariously at the top to enable me to see the nestlings up close. What was I doing? I was so totally involved, my social life suffered greatly but, somehow, I did not notice.

It was an obsession and, as in any obsession, one did not ask rational questions about the need and sense of it all. In those four years or more, sleep declined to 4 h maximum a night and yet it all seemed terribly exciting, urgent, and it felt right and the only thing to do. Via the trigger of the magpie’s speech, I had found a passion in life so great that none of the usual questions about feasibility, value or career seemed to matter in the slightest. There were no forward plans made outside the next “urgent” magpie project. Magpies led me into the bird world.

In almost all this time of my research, I was on my own in the field in the company of magpies. They had allowed me to observe them at close quarters and I had learned to distinguish between them by their wing markings. which, incidentally, are individually different and remain an individual attribute for life [33]. I began to see how they lived their daily lives, their struggles, their ingenuity and their very considerable problem-solving abilities and methodical ways.

Without hesitation, I took a job as a junior research fellow, then finished this second PhD and was gradually promoted through the ranks (again), first being paid entirely through grants. I had even lost my university employment but got it back once promoted to a full professor again, in animal behaviour now, and what followed was an explosion of creativity, of research papers and books just tumbling out and, of course, by then research findings had also led me to other bird species, their skills, communication, cognitive, and problem-solving abilities in an evolutionary and environmental context. None of my work has ever required handling birds, tagging, or provisioning them. As in Jane Goodall’s research with the Gombe chimpanzees, it was patience (for slow habituation) and observational skill, scoring behaviour from a distance. Such research takes much longer that laboratory research because one cannot control all the variables and sometimes, the behaviour one is after, simply does not occur. For specific questions, field research can be done using some “props” such as playbacks of their own alarm calls to test referential signals [34,35] for instance, or placing taxidermic models in the field for testing both communication and strategies of predator defence [36,37]. To this day, I think of avian research as a green meadow into which one can run and feel free and share life with those birds that are part of this continent.

Perhaps this magpie event is an odd tale about bird research. It is such a rare event when one has found one’s niche, a sense of belonging into that niche at a point where motivation is not explicable in career terms or in having to dream up new projects but where natural curiosity and delight in what one is doing and finding create their own momentum. But perhaps it is time now to analyse what these events actually mean and what they can perhaps tell us beyond personal experience.

## 3. Discussion

The first two cases presented here show certain overlaps and Case 3 is an important disjuncture. I will discuss cases 1–2b together because they represent variants of the theme of interactions with great apes. Case 3 will be discussed separately as this might well fall into an entirely different set of discourse, one that we probably have not had but should have, as will become clear.

### 3.1. Cases 1–2b: Chimpanzees and Orang-Utans

Gary Ferguson [38] wrote a book *Opening Doors* and told the true story of Carole Noon who, inspired by a lecture by Jane Goodall, turned into a passion to save chimpanzees from biomedical research, the entertainment industries and the pet trade. And she succeeded, founding the “Save the Chimp Sanctuary” which has grown into the largest primate sanctuary in the world. There are obviously more than a handful of people who feel a deep sense of obligation to care for the animals we, as people, have chosen to subjugate and use in whatever manner we wish. We even keep them for ourselves, regardless of the cost to the animals removed from their own species, families, and natural environments.

However, what happened in the case of James Mahoney, was not based on a deeply held ethical position. This was most likely the case for Carole Noon. She was being persuaded by a lecture—a reasoned and empathetic argument that chimpanzees deserved our attention and better treatment than they were receiving from humans.

James Maloney made it very clear in his address at Wisconsin University that it was the touch on his arm that jolted him into a “parallel universe of himself” (his words). True, he had been a veterinarian and thus had been trained to improve the health and well-being of animals, not to inflict suffering and pain and that “other self” had to be suppressed in order to serve another, contradictory but equally noble purpose, and that was to benefit humans and improve their health, save human lives and accept the chimpanzee sacrifice—these were the options and that goal, to find a cure for AIDS and prevent the horrific deaths of thousands was in line with the Hippocratic oath: First, “do no harm” (Latin: “primum non nocere”), or now more generally seen as a concept of non-maleficence. And the other half of his psyche, trained as a veterinarian, demanded the same moral position of doing “no harm” which, by accepting the job as he had, constituted the most basic breach of the rule of non-maleficence.

Incidentally, for all the well-publicised rescue of these chimpanzees, several research centres continued to use chimpanzees in biomedical research and the National Institutes of Health (NIH) continued to spend about US$12 million a year caring for the chimpanzees it supports (totalling 734 in 2011) and even suggested to recall 186 semi-retired chimps back into active research—all this happened still in Mahoney’s life-time [39].

Whether or not he managed to suppress one for the greater good of the other effectively, he did not say, or whether he changed his view about human relationships to chimps, he also did not mention. Whether or not the chimpanzees stayed in the research facility or were moved to another environment, never actually required a shift in control over the chimpanzees. Their fate lay squarely in the hands of human organisers, a fate that could also change again, indeed as it did for some. Chimpanzees were expensive goods, merchandise and useful items for research. To save money, females were impregnated every year and the one year-olds removed from the mother in order to live an equally miserable life as test animals, as mentioned before.

The chimpanzees had been trained to extend their arms outside the cage bars to receive injections so he did not usually look into the face of the chimpanzees. There was also the issue of the stench in the experimental rooms because the cages dangled mid-air, with bars as a floor, meaning the chimpanzees urinated and defecated onto the floor of the room and no manner of cleaning could quite remove the unpleasant and permanent stench. It was not a room in which the human staff wanted to linger and any contact with the chimpanzees was normally brief and professional.

The chimpanzee who touched Mahoney intercepted that routine. We know from decades of research, that all great apes possess a complex gestural repertoire often seen as pre-speech acts (chimpanzees [40,41,42]; bonobos [43,44]; orang-utans [31]). When sign language was taught to apes it also became clear that all great apes are capable of displaying an understanding of human words, objects, commands, and numeracy (chimpanzees [45]; bonobos [46]; gorillas [47]; orangutans [48]). Incidentally, all these abilities have later also been shown in birds, e.g., by Alex, the African grey parrot [49,50]. In primates, this abstract ability of being able to deal with symbolic representation of language and thus complex cognitive processes, has led to more detailed investigations both of behaviour and of the structure and function of the primate brain [51,52,53] and about their pre-speech acts among each other and within their own species.

More recent research on chimpanzees by Kim Bard and colleagues [54] has for the first time taken a “bottom up” approach, observing just one gesture: Touch, and then recorded location of touch and possible meanings of this gesture. They found that this single gesture had 36 different forms, was directed to 70 different target locations on the body of social partners, and occurred in 26 different contexts.

Touching of the arm (as a specific location) occurred largely only in two contexts, one as a contact gesture generally and in a grooming/affiliative context. Touching for comfort was only observed and initiated by juveniles in 4% of all observed cases of touching while in adult initiated touching this was a mere 1% [54]. It is possible therefore that the gesture of touch was misinterpreted by Mahoney and most others who saw or were told of it. But in human culture, a squeeze or gentle touch of the arm by another can have decisive cultural meaning as a gesture of understanding, sharing, consoling the person at a time of grief or inner conflict.

The power of this gesture by the chimpanzee, especially after some time of Mahoney’s absence due to his illness, was no doubt understood by Mahoney in the manner in which it is usually understood in western culture (whether this is the meaning the chimp wanted to convey is yet another matter). It had an instant and electrifying effect on Mahoney and changed his life and that of hundreds of chimpanzees almost at once. In the obituary published online in *Animals 24-7*, James Maloney was described as “the Oskar Schindler of laboratory primates,” after the German industrialist who saved 1200 Jews from Nazi death camps in 1944–1945 by routing them to safety through employment at his factories” [22]. However, there is something wrong in this analogy: Mahoney had to switch sides from tormentor to saviour. Schindler, for all his faults, never had to. One may read his story as one of conflicting moralities, perhaps even double-standards, but most of all, what was important to convey here is that the gesture of touch, as a widely used human gesture, was the reason for Maloney’s change of heart. He had interpreted this touch as an act of an individual with feelings and the capacity for empathy, perhaps not consciously so, but one he thought he understood. He said that he thought in that second that chimpanzees were sentient beings and that he had inflicted untold suffering on them.

Importantly, chimpanzees were known taxonomically to be the closest ancestor of humans and had been studied for many years, not so much for their own sake as for understanding the origins of humans. This was initially Jane Gooddall’s assignment too. That she turned it into something else and something much more important (understanding chimpanzees for their own sake and chimpanzee societies in their own dynamics), is a different matter [40]. To this day, documentaries on chimpanzees proudly mention that chimpanzees have over 97% of their DNA in common with humans. True, we have about 98 percent of our genes in common with chimpanzees and 97 percent with orang-utans [55]. While such results sounded like solid evidence of even more than just taxonomic relatedness at first, the genome mapping when it was completed for mice in 2012 revealed something else. Results showed that humans share 99 percent of their genes with mice [56], i.e., more than with chimpanzees. Apparently, we even share over 60% of our genes with bananas. How is this possible?, many asked. It is so because it depends which genes are actually expressed and further because the building blocks of all life on the planet are made of the same basic biochemical components, if with different constraints and expressions. In popular culture there has been a profound misunderstanding of the role of genes [57].

In case 2a, Abbi’s gesture, raising arms to indicate that she was about to climb up on me, was also a gesture initiated by the orang-utan. Here the situation was different. Mahoney inferred that the chimpanzee consoled him (attended to his needs), which would no doubt be seen as a profoundly empathetic act. In the case of the orphaned orang-utan, it was the opposite. Abbie was most likely driven by her very basic need to be close to a mother figure, a need that had not been met since her mother died. Orang-utan infants stay and sleep with their mother in the same nest and travel close to her through the canopy until they are about seven years old—even at the age of five, a close cuddle is still needed and only eventually a physical distance is created. Abbi was only 4 years old [31].

However special and deeply moving these meetings with Abbie had been, they remained singular exchanges, did not cause major upheavals or fractures in her or my life and did not result in anything dramatic as it did in the sensational events surrounding James Mahoney and even Carole Noon. Mine were moments, precious and never forgotten, but what was their possible meaning? That we are more alike than I had thought? No, I simply had not thought about animals at all before and had almost entirely lacked any experience with any mammals or even vertebrates.

Hence, these events did not *change* my thinking but became the template for thinking about animals. My introduction to Abbi was also my introduction to animals, to a whole world “out there” that I had not seen or noticed, not thought or cared about in the least. It was profound because of a sudden awareness that I had shut out half the world and its precious live contents, not because of fear or dislike but because they simply had not featured.

My experience with Simbo was on a very different plane. It reminded me of Val Plumwood’s writings. Plumwood argued against the “hyperseparation” of humans from the rest of nature and what she called the “standpoint of mastery”; a reason/nature dualism. She died in 2008 but even beyond her death retained the status of an iconic ecofeminist and had locally at least something of a cult following. The article that impressed me most at the time was called “Being Prey” [58] recounting when she was attacked by a crocodile and endured and survived several death rolls and being plunged under water… I remembered her story not just because of her terrifying experience but because of her gratitude to have survived which “cast a golden glow” over her life and her insistence that no shooting party should be sent out to randomly destroy crocodiles.

Predatory animals do what they do without hatred and usually without a sense of vengeance or meanness. They either want to eat—and we might become food—or they want their territory left undisturbed from invasion. Most wild animals know that humans are dangerous and will occasionally make pre-emptive strikes but nobody has yet shown that they are purposely vengeful. As I wrote recently, in response to an article arguing that some animals are like humans and should be considered so, I replied that this would not be desirable because humans have a dark side. Mostly only humans have large-scale warfare and spend much time and imagination in devising ever more terrifying weapons to kill each other. They kill animals even if they do not need to and just like it as a “sport”, and only humans, exclusively humans, have invented torture and have been extremely inventive in designing instruments and tactics to this end [59]. While I have no sympathy at all for this violent side of humans, there is also the opposite. Some animals are simply larger than humans. Even if they do not want us as prey, a moment of anger, fear, or aggression may well kill a human when facing off one-on-one, be this a bull, a wild boar, an elephant, or an orang-utan.

Potentially, Simbo could have thrown me about like a rag doll for defying him, the unchallenged king of this patch of forest and not doing what the other orang-utans, females and juveniles alike, had already done: Scamper. Instead, I was standing my ground and he had responded by very swift and seemingly aroused if not angry movements towards me. I had no defences whatsoever against his physical strength and the only strategy I could think of was to slip into accepted orang-utan submissive behaviour, head turned away, face half down and eyes averted and wait. Any movement on my part could have been interpreted as provocation. He chose not to “teach me a lesson” but disappeared again in the direction from where he had come. It was a warning though and one might not be so lucky the next time. The “never do this again” thought was firmly implanted in my mind.

Importantly, this encounter has been included here simply to dispel any notion that wild orang-utans are toy things for us and that the only stories one can tell about them are in the cute and cuddly category, nor give the impression that they are “so like us”—even if, in many ways, we probably are, certainly biologically. Great apes may be similar to us and have been of increasing interest to the public but often this interest is linked to expectations of them having to be nice and accommodating and preferably both.

In order to have our respect, animals should not be required to “live up” to such expectations or having to act as entertainment for human benefit. Having to be exceptionally cute or entertaining or at least well-behaved is setting the bar high, not achieving it may mean a loss of interest and even a loss of sympathy or good intention by people. An animal considered “dangerous” usually gets shot.

Alternatively, we take them into our fold and explain their needs as we understand and feel them. As Fouts and Mills wrote in the book *Next of Kin*, 1997 [60], presumably to gain support for the point of view that the chimpanzees used in experiments, shows and I quote them:

“All of the chimps felt the same pain of loneliness and a terrible fear about their strange new surroundings. Each of them had the same deep need that you or I would for the comfort of physical contact and affection. That was the tragedy of putting these social creatures in the solitary cages that dangled above the floor” [60].

The passage is declaring in the same breath that chimpanzees are like “you and me” and then calling them “creatures” as a safe way to re-establish that they are not the same but animals, putting a safe distance between humankind and apes…

It is a view firmly entrenched within human exceptionalism, a way of graciously portraying a tolerant attitude to animals and/or expressing views of somehow “lifting” the animal similar to us into human spaces, meant to portray an act of human magnanimity. Indeed, such acts negate what has been called “selfhood” in animals. They are indeed “subjective others” and not just the objects of anthropomorphic projection [61]. The similarity may be self-reflective and have nothing or little to do with the actual “subjective other” that they see in front of them.

Hampton [62] who has been critical of overinterpretation of behaviour in apes and corvids, argued that “When we leap to embrace superficial similarities among species, […] we may foist our cognitive preconceptions on other species”. Indeed, similarities between humans and great apes may at times have the opposite effect and lead to a thorough misreading of a situation, a mood or an intention by the primate.

Hampton also argued his case in the context of laboratory tests and demanded “experimentally rigorous analysis” [62]. That may be true but, in many contexts, I fear, that no matter how rigorous the experimental design is, there are two variables in captive experiments (to which Hampton made reference) which are all too often downplayed or brushed aside.

One is that the experience of prolonged captivity may not change but distort, simplify, and even enhance traits that are typically not displayed by a species in the natural environment.

Second, behavioural experiments, benign as they may be (compared to those that may do physiological harm), are still enforced upon the other and can be highly contrived, leading to highly contrived results for several reasons and not all can be mentioned here. They may have parameters and suppositions that are irrelevant in terms of the ecological niche and the social requirements that a given species may occupy or need in the wild. For instance, they may require a particular kind of memory or skill that the experimenters want the test subject to display. In captivity, especially in species that are able to learn what is required of them, can adapt and will perform tasks that are entirely uncharacteristic of the species in their natural environmental. One standard design for testing cooperative behaviour, to give just a few examples, is to ask primates to use a rope attached to a box full of food that is too heavy for one chimpanzee to pull towards itself. A second chimpanzee needs to pull at a second rope and pull simultaneously and in the same direction as the other one. Chimpanzees need many training sessions for this and even then, the outcome is not always successful [63]. Some older experiments (1961) engaged in reinforcement task experiments that asked the chimpanzee to press levers for a pellet reward—up to 450 in a single day. The experimenter wrote without apparent guilt or second thoughts that by the end of 50 days (sic!) the subject responded correctly to between 98 and 99.8% of the presentations [64]. This study and the previous study by Rohles et al. 1961 were explicitly conducted to measure higher cognitive functioning in animals [65]. We have moved a long way from mindless to mindful experiments in the meantime. However, the problem remains that such artefacts of experimental design or even positive outcomes that expose remarkable behaviours in captivity, may not at all or rarely occur in the wild and therefore tell us relatively little about the dynamics and the evolutionary path that certain animal groups have taken.

To demonstrate the point, and to lead into the next discussion point on birds, I will give just two examples. Irene Pepperberg’s work with Alex (*n* = 1), the African grey parrot, *Psittacus erithacus* [49] has spear-headed a great deal of cognitive research in parrots and one needs to be indeed grateful for her work in having opened doors for asking different questions and for proceeding in experiments by turning the subject into an active participant of specific studies, be this on linguistic or numeral comprehension [50]. Alex was able to reply to questions (in words that he had been taught) and he made correct statements in counting objects (up to four). However, when observations were made of wild African grey parrots in their natural environment, it took years to discover some limited mimicry in African grey parrots found eventually in Botsima, in the Salonga National Park, Zaire [66,67]. In a set of more recent laboratory studies on Goffin cockatoos, *Cacatua goffiana*, one laboratory devised a series of scientifically-flawless experiments showing impressive tool using and tool making abilities in this species [13,68,69] and then wisely proceeded to study the species in the wild (on the island of Tanimbar, off the north east coast of Australia) and found that they do not use tools in the wild at all [70]. It depends, of course, what it is that we want to establish by testing animals and what questions one asks.

### 3.2. Discussion: Case 3

This push and pull between suggesting that animals may be sentient beings and at the same time reaffirming that humans are “unique” and exceptional is a contradiction we see played out regularly.

The cognitive revolution, probably starting with Donald Griffin’s work on the prospects of a cognitive ethology in the late 1970s [71], led students of primate social behaviour to focus on new kinds of questions that, as Peter Marler wrote in 1996 [72] rarely arise in more traditionally-oriented studies of the social behaviour of birds. There had been early beginnings with the studies by Koehler and Thorpe in the 1940s and 1950s but they had gone out of fashion. Bird cognition, also as a comparative discipline, really only started with articles like Peter Marler’s “Are Primates smarter than birds” and Irene Pepperberg’s work with Alex already mentioned, only slowly making inroads into the specific aspects of avian life histories [73]. In the last ten to twenty years the comparative work has culminated in a range of very fertile findings in neuroscience, neuroethology, biology, and endocrinology combined with behaviour. Finally, scientific results have convincingly shown a previously-unimaginable richness of diversity of abilities and mental capacities in birds. It was discovered that some species had phenomenal memory, others were capable of planning, problem-solving, tool using, and innovation too numerous to cite here.

The findings have gradually dispelled the notion that avian species lag far behind the primate line in cognitive abilities as well as in the affective domain. Avian cognition is now a separate field of study and this field has done a good deal to break down the Aristotelian idea of *scala naturae* and claim the uniqueness of human characteristics. Clearly our superficial judgements were once also guided by superficial similarities and difference of appearance and that judgement was persuasively supported by philosophers such as Descartes that birds cannot think and only act like little automatons. What happens to our empathy and enjoyment when “the other” looks nothing like us and we have already pre-empted their own selfhood by believing that they have no thoughts or emotions of their own?

One can think of many differences, of course, between birds and great apes or humans and they do not need to be enumerated here. One of the highly-important differences is the head. First, birds do not have the neocortex of mammals, the very area of the brain thought to be essential for any thought processes in the forebrain. Secondly the size: The tiny heads of birds compared to mammals and primates apparently and “obviously” showed that birds have minimal cognitive capacity and function entirely on genetically preset behaviour. One bombshell was dropped in 2016 when scholars from Prague, Vienna, and from Brazil started measuring not overall brain size but, by using an isotropic fractionator, could actually determine numbers of neurons in specific brain regions. They found, and I quote:

“That the brains of parrots and songbirds contain on average twice as many neurons as primate brains of the same mass, indicating that avian brains have higher neuron packing densities than mammalian brains. Additionally, corvids and parrots have much higher proportions of brain neurons located in the pallial telencephalon compared with primates or other mammals and birds. Thus, large-brained parrots and corvids have forebrain neuron counts equal to or greater than primates with much larger brains” [74]. Obviously, attitudes that suggests that birds are like “automatons” is no longer tenable and, as an aside, it is incomprehensible to me why anybody ever entertained such notions at all. However, now that it is finally conceded that birds can think, the question also had to be asked whether they can express emotions.

A bird’s head is so different from that of a human or primate, we are likely to read nothing from it. And yet, that too is a false first impression. The argument that we know what “they” think in great apes and even about other monkeys is often subconsciously related to the face—humans and primates have the same facial muscles, at least the great apes and a few others that have been tested. Apes and monkeys have frontally-placed eyes, have similar vision and hearing to humans, and can move their faces, including eyebrows, mouth, and sometimes even the tongue (in cases of disgust) in order to express a particular state or emotion. However, as Morimoto and Fujita were still able to say in 2011 [75], facial expressions were mainly studied in chimpanzees because, for a long time it was doubted that platyrrhine and strepsirrhine primates had facial expressions differentiated enough, or their social system complex enough, to be capable of using facial expressions as part of their communication system. They tested capuchin monkeys (*Cebus paella*) and showed that capuchins, a South American species, modified their behaviour in accordance with facial expressions of a conspecific [75]. We undertook a study of facial expressions in common marmosets, *Callithrix jacchus* (we had our own and well-established colony of marmosets on the university campus), also at home in South America. We discovered that they displayed an unexpected variety of facial expressions and that cage mates responded strongly to facial expressions of fear and pleasure when the images were shown on a screen behind a food bowl full of their favourite food: A displayed fearful face of a cage mate kept them away, a happy or pleased face made them approach the food bowl and take the food [76]. In the last decade, researchers working on facial expressions have developed a coding system, now called Facial Action Coding System (FACS) and have done so species by species to avoid reading meanings into facial expressions or using different criteria than another researcher (orang utan [77]; chimpanzee [78]; Macaque [79]; and gibbons [80] in order to have some standardised facial expressions that can be used by different researchers [81]. Facial expressions and the eyes are clearly important to humans as indicators of mood or intention and eyes have often been called the window to the soul.

By contrast, birds have even been doubted to have faces and apart from my research, speaking deliberately about faces in birds, there has only ever been a rare mention of a bird’s face and then by only two researchers, Richard Andrew and Pat Bateson, both of whom were friends to the end of their lives.

**Figure 4:**
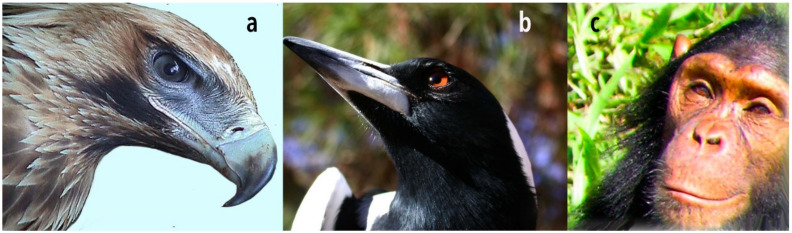
Caption: The heads of (**a**) a juvenile wedge-tailed eagle (*Aquila audax*) and (**b**) of an adult Australian magpie (*Gymnorhina tibicen*). The strong beak of the eagle and the eyes set deep under a protruding brow, make the bird appear dangerous and give the eyes an almost hostile appearance; and the powerful beak is often misjudged as a weapon. That is mostly incorrect. The beak is so strong in order to tear open mammalian hides, not for fighting and biting. The business end and the “weapon” are its talons. The magpie, even with its soft brown eyes, still has a remarkably strong beak and, in this species, the beak is indeed used for fighting and for administering punishment to intruders or unruly youngster. Neither face may give us readily identifiable cues about mood or intentions. By contrast (**c**): A face of a chimpanzee in the Ngamba Sanctuary in Uganda seeing a friend approach—a relaxed face (squinting only because of the sun) but there is a faint smile that we can readily recognise (Credit **a**,**b**,**c**: G Kaplan).

The reasons why most researchers shy away from giving birds a face are understandable. There is nothing in a bird’s face with which we can spontaneously and easily identify or from which we could read emotions (Figure 4), so it seemed. The eyes are not facing forward and have little mobility, an anatomical feature that could be misread as a hostile stare, especially in eagles with their protruding brows. We further rely on mouth and forehead movements of muscles as key indicators of another person’s moods. But for birds generally, the “face” is fixed (Figure 4) and not a source to “read” intentions and moods. At least this is what was believed.

As late as 2013, the idea of facial expressions in birds was only politely conceded. For instance, Waller and Micheletta [82] said:

There are a few compelling examples that could be considered to be facial expressions (in a sense). The facial components of birds’ heads are not particularly mobile and they do not have facial muscles [82].

While this is true as far as major facial musculature is concerned, it is not true that birds do not have facial expressions. It is a constant problem of lack of cross-communication between researchers who have specialised in specific animal groups. Among scholars of mammalian behaviour there are dog people, cat people, horse people, bat people, and primatologists and then there are ornithologists. Some of these divisions are inevitable when working in different classes such as Class Mammalia and Class Aves. Those scholars who study primates may not speak with scholars of avian species, let alone read their work, and vice versa. A bridge has now been created by comparative studies of cognition in animals [83,84,85,86]. Clear evidence of this failure to cross communicate or what had been unkindly called “chimpocentrism” or “primate-centrism” by scholars working outside the field of primatology and now also in cognitive functions in invertebrates [17], and even within it, also perhaps implying a borrowed attitude of human exceptionalism. The view quoted here by Waller and colleagues who had done such an excellent job in driving forward research of primate facial expression, clearly relied on an anatomical source that only mentioned major muscle groups without considering the advances in avian research.

The head of birds is subdivided into several regions. Ornithologists developed the map of the head with its many different areas for one simple reason, and this is for the purpose of species identification by plumage colour and patterns. For instance, in the family of honeyeaters (Meliphagidae), a large and diverse family, which includes scores of species named honeyeaters but also the Australian chats, friarbirds, wattlebirds, and miners, some species differences are most-easily- or only- identifiable by patches of colours and patterns on the head and exactly where these patches are located.

The subdivision of the head into specific segments also proved worthwhile now for describing feather movements of the head and neck and the back of the head as well.

In 2003, a decade before the statement by Wallers and Micheletta, Homberger and de Silva [87] had published a paper identifying the mechanical forces in the feather-bearing skin (integument) for the entire feathered body, including the head. They described in detail the biomechanics of an integumentary musculature in birds [87] and showed that this musculature, consisting of smooth erector feather muscle; smooth depressor feather muscle; and smooth apterial muscle, allowed independent feather movement up and down of even small areas of the head. Some birds, namely cockatoos, have moveable crests as well, used readily in communication. Most parrots have an elaborate system of communicating their state or intention by feathers alone. In cockatoos, the position of the crest can indicate play readiness (fully forward), alarm (half-mast), or anger (erect), and can be accompanied by body movements such as head bobbing or wing flapping (play readiness), arching of neck (anger), and also by simultaneous vocalisations (for an extensive coverage of these issues, see Kaplan 2015 [14].

Facial expression is achieved by movements of the beak, by independent positioning of feathers below or above the beak, on the ear coverts, on top of the head (the crown), at the nape of the neck and, in some species, also by moving the feathers above or below the eyes independently of other feathers. Magpies do not have crests and relatively little feather movement on the head but rising anger shows up as slight feather erection below the eyes and is usually accompanied by sharp short vocalisations [33].

In recent research, the role of colours in the communication of emotions between birds has lead to surprising discoveries. Changes in facial colouration are well-known in the human context [88] but these are entirely new discoverie in birds. For instance, blue and yellow macaws (*Ara ararauna*) not only have facial displays but do so by spontaneous colour changes in their face, both at the bare skin patch (“blushing” turning a hue of pink) and changed feather positions dependent on the birds’ social context and activity. These findings have opened the door for further investigation into the potential importance of facial expressions in close range communication in birds [89].

Birds also have open mouth displays or, rather, open beak displays, which, together with other body signals, are used in fear or threat displays. Head feathers, depending on their location and combined raising or lowering of feather in the nape of the neck or of the whole body, as has been shown in threat displays of tawny frogmouths, *Podargus strigoides* [90] can add up to at least 20 different combinations which have to be contextualised.

Equally, vocal mimicry has often been dismissed as a mindless act in birds because no general or specific function could be found [91]. However, there is at least one function of mimicry that may well be universal, namely as the first stage in vocal learning. In Australian magpies, these first babblings follow trajectories of gradual sound identifications and vocal shaping, even following similar stages as in human infant babblings [92].

Finally, researchers in comparative affective neuroscience have identified the hormonal framework that characterises human, primate, and also, as is now known, avian systems. Indeed, birds have the same package of hormones as humans and other mammals. In both primates and birds, simple behavioural mechanisms such as allopreening (preening someone else) and roosting closely together are linked to oxytocin.

Monoamine neurotransmitters, such as dopamine, noradrenaline, adrenaline, and serotonin also have roles to play in regulating emotions. Serotonin (5-hydroxytryptamine) is a monoamine neurotransmitter that controls mood and certain functions in the brain. In humans, low levels of serotonin have been associated with depression and normal levels have an impact on sleep. Other actual biological functions, while complex and multifaceted, have been shown to include modulating memory, learning, cognition generally, and even sexual appetite. Serotonin is often thought of as alleviating stress and promoting relaxation. Dopamine (3,4-dihydroxyphen-ethylamine), partly with its own network, plays important roles in executive functions, motor control, motivation, arousal, reinforcement, and reward. Trying to tease out the role of the opioid receptors and serotonin gets complicated because of their interactive role. They also work to stabilise the immune system and have an effect on sleep. How brain pathways and the dynamics of neuropeptides evolved to generate complex social behaviour, as Chakraborty and Jarvis (2015) rightly argued, remains an enigmatic but fundamental question in biology [93].

Equally fascinating and important is our present understanding of the evolution of the brain. It was not too long ago that we believed that small brains, and especially those without a neocortex, could not possibly have any significant cognitive power. This is a view that has been corrected now, not only because neuroscience had entered the fray about cognitive abilities in birds [94] but, at the same time, it became common knowledge via the development of the computer and the increasing technology for storage of vast amounts of memory in small spaces, that overall volume may not be the determinant for the amount of memory that can be stored. We have also since learned that birds have strong likes and dislikes, have personalities, make decisions, are innovative, can bond strongly with partners [95], and have many other qualities that more than suggest that amongst Class Aves, there are species of substantial complexity.

Still, birds had a tough time emerging from the cloak of moral indifference that Descartes had put over all animals because, he argued as mentioned before, they have no feelings, no thoughts, no minds of their own and were merely instinct driven automatons. Hence humans have no moral responsibility and can do what they like with them. And this belief has stayed with us and with science well into the middle of the 20th century. Kleiner [96], in reviewing a book by Steven Wise *Drawing the Line*, remembered his high school biology classes that still taught that animals are mere instinct driven automatons, and that notions of intelligence, reasoning, and even emotions were a mark of poor science and of anthropomorphising. Val Plumwood, in her review of Raimond Gaita’s book *The Philosopher’s Dog* [97] reminded us that this posturing is still with us today. According to Gaita, moral status can still be denied to animals because they lack the human equivalent of “fine-grained forms of individuality” and, even more depressingly, because “they” (meaning all animals) “are replaceable, unlike human beings, who are unique and irreplaceable and ‘precious as nothing else we know in nature’ (p. 199)” and this was said and thought in the 21st century and restated in a reprint of 2017 [98]!

## 4. Conclusions

In summary, bird self-expressions, in some species, perhaps not in all, may be as varied as those across the primate line. The common ancestor between birds and humans is a distant 300 million years away, as compared to a mere 5–6 million years in primates. Their abilities, some of which are now known to be equivalent to chimpanzees [74], still stun and surprise.

Remembering that my interest in the magpie was roused when it offered me a bridge by shouting outside the window “I have got dinner for you”, my response was clearly guided by the familiarity of the utterance. In all the positive and life-changing experiences of animal encounters described in this paper, it perhaps needed a signal understandable in the realm of human existence (touch, embrace, human words) for a transition in thinking about animals to occur. That these instances would surprise is a reminder that we are living in our own society and perhaps, at best, can expect to think in a biocentric anthropomorphism [99] and at least an animal-centred anthropomorphism. Field work, as I do, can certainly be that: Entering their world and doing it as gently as possible with slow habituations as Jane Goodall had done with her chimpanzees [40]. I consider her work so invaluable because she made a substantial contribution to understanding chimpanzees on their own terms, without changing their own society and without dominating their lives. Giving other living organisms the right to be, precludes judgements of worthiness and replaceability [98,100]. An animal should not have to prove that it is sentient or capable of things that its evolutionary niche may not even require. However, we, collectively, have always underestimated the capacities of animals, cognitively, affectively, and socially. The transformative experience of hearing this magpie speak was not just because of the recognisable human signal. It was much more. In one big jolt, this bird, charming, inquisitive and perceptive, was “telling” me of my thoughtlessness. In this human-centred cocoon of my world, the bird made me realise the utter marginalisation to which I had relegated birds in particular. The magpie made itself centre stage for that moment and I have made the magpie and other birds centre stage ever since. Still, no matter whether naïve or conscious anthropomorphism, we cannot help but see the world from our perspectve even if we may be capable and successful, to some extent, in attempts of taking an animal’s perspective. Moreover, we are embedded in a tradition of an Aristotelian *scala naturae* which is worth fighting against both for scientific rigour and for living with respect for the natural world. Finally, as Williams et al. [19] had put it so well recently, and I quote them directly:

“Most researchers advocate being mindful of parsimony and avoiding assuming complexity. Applying such views to comparative affective science, it is crucial to restrain from presuming that affective processes in humans are complex and to consider the degree to which the most parsimonious explanation may be the best explanation”.

Indeed, not all processes in humans are complex. To have turned this view around (i.e., instead of applying to research on animals, to apply the same caution to human (self)) assessments is a valuable step in giving up a position of human exceptionalism.

## References

[1] Pickering A. (2008). Against human exceptionalism. XTR Workshop-250208 ′What Does It Mean to Be Human’.

[2] Larson S.G., Jungers W.L., Morwood M.J., Sutikna T., Saptomo E.W., Due R.A., Djubiantono T. (2007). Homo floresiensis and the evolution of the hominin shoulder. J. Hum. Evol..

[3] Evans P.D., Mekel-Bobrov N., Vallender E.J., Hudson R.R., Lahn B.T. (2006). Evidence that the adaptive allele of the brain size gene microcephalin introgressed into Homo sapiens from an archaic Homo lineage. Proc. Natl. Acad. Sci. USA.

[4] Mather J. (2019). What is in an octopus’s mind?. Anim. Sentience.

[5] Chapais B., Kappeler P.M., Silk J.B. (2010). Mind the Gap. Tracing the Origins of Human Universals.

[6] Shapiro G.L. (1982). Sign acquisition in a home-reared/free-ranging orangutan: Comparisons with other signing apes. Am. J. Primatol..

[7] Vallortigara G. (2012). Core knowledge of object, number, and geometry: A comparative and neural approach. Cogn. Neuropsych..

[8] Vallortigara G., Regolin L., Chiandetti C., Rugani R. (2010). Rudiments of mind: Insights through the chick model on number and space cognition in animals. Comp. Cogn. Behav. Rev..

[9] Spelke E.S. (2000). Core knowledge. Am. Psychol..

[10] Finlay B.L. (2019). Human exceptionalism, our ordinary cortex and our research futures. Dev. Psychobiol..

[11] Marino L., Connor R.C., Ewan Fordyce R., Herman L.M., Hof P.R., Lefebvre L., Lusseau D., McCowan B., Nimchinsky E.A., Pack A.A. (2007). Cetaceans have complex brains for complex cognition. PLoS Biol..

[12] Taylor A.H. (2014). Corvid cognition. Wiley Interdisc. Rev. Cogn. Sci..

[13] Auersperg A.M., von Bayern A.M., Weber S., Szabadvari A., Bugnyar T., Kacelnik A. (2014). Social transmission of tool use and tool manufacture in Goffin cockatoos (Cacatua goffini). Proc. R. Soc. B Biol. Sci..

[14] Kaplan G. (2015). Bird Minds. Cognition and Behaviour in Australian Native Birds.

[15] Bensky M.K., Gosling S.D., Sinn D.L., Brockmann J.H., Roper T.J., Naguib M., Mitani J.C., Simmons L.W. (2013). The world from a dog’s point of view: A review and synthesis of dog cognition research. Advances in the Study Behaviour.

[16] Byrne R.W., Bates L.A., Moss C.J. (2009). Elephant cognition in primate perspective. Comp. Cogn. Behav. Rev..

[17] Cimatti F., Vallortigara G. (2015). So little brain, so much mind. Intelligence and behaviour in non human animals. Reti Saperi Ling..

[18] Zhang J.W., Piff P.K., Iyer R., Koleva S., Keltner D. (2014). An occasion for unselfing: Beautiful nature leads to prosociality. J. Environ. Psych..

[19] Williams L.A., Brosnan S.F., Clay Z. (2020). Anthropomorphism in comparative affective science: Advocating a mindful approach. Neurosc. Biobehv. Rev..

[20] Mendoza S., Box H.O., Hutz R., Fragaszy D., Tuttle R.H. (1996). Proceedings of the xvith congress of the international primatological society. Int. J. Primatol..

[21] Rudman L.A., Ashmore R.D., Gary M.L. (2001). “Unlearning” automatic biases: The malleability of implicit prejudice and stereotypes. J. Person. Soc. Psych..

[22] Clifton M. James Mahoney, DVM, “the Oskar Schindler of Laboratory Primates,” Dies at 77. animals24-7.org.

[23] Martin D. (2005). LEMPSIP. The New York Times.

[24] Kaplan G., Kaur A., Metcalfe I. (1998). Economic Development and Ecotourism in Malaysia. The Shaping of Malaysia.

[25] Haraway D. (2008). When Species Meet.

[26] Pratt M.L. (2008). Imperial Eyes: Travel Writing and Transculturation.

[27] Wilson H.F. (2019). Contact zones: Multispecies scholarship through imperial eyes. Environ. Plan. E Nat. Space.

[28] Parreñas R.J.S. (2012). Producing affect: Transnational volunteerism in a Malaysian orangutan rehabilitation center. Am. Ethnol..

[29] Kaplan G., Rogers L.J. (1994). Orang-Utans in Borneo.

[30] Kaplan G., Rogers L.J. (2002). Patterns of Gazing in Orangutans (Pongo pygmaeus). Intern. J. Primat..

[31] Kaplan G., Rogers L.J. (2000). The Orang-Utans. Their Evolution, Behaviour, and Future.

[32] Rogers L.J., Kaplan G. (1996). Hand preferences and other lateral biases in rehabilitant orang-utans (Pongo pygmaeus pygmaeus). Anim. Behav..

[33] Kaplan G. (2019). Australian Magpie.

[34] Kaplan G., Johnson G., Koboroff A., Rogers L.J. (2009). Alarm Calls of The Australian Magpie (Gymnorhina tibicen): I. Predators Elicit Complex Vocal Responses and Mobbing Behaviour. Open Ornithol. J..

[35] Kaplan G., Rogers L.J. (2013). Stability of referential signaling across time and locations: Testing alarm calls of Australian magpies (Gymnorhina tibicen) in urban and rural Australia and in Fiji. PeerJ.

[36] Kaplan G. (2011). Pointing gesture in a bird-merely instrumental or a cognitively complex behaviour? Special Issue ‘Animal Cognition’. Curr. Zool..

[37] Koboroff A., Kaplan G., Rogers L.J. (2013). Clever strategists: Australian magpies vary mobbing strategies, not intensity relative to different species of predator. PeerJ.

[38] Ferguson G. (2014). Opening Doors: Carole Noon and Her Dream to Save the Chimp.

[39] Wadman M. (2011). Animal rights: Chimpanzee research on trial. Nature.

[40] Goodall J. (1988). In the Shadow of Man.

[41] Krause M.A., Fouts R.S. (1997). Chimpanzee *Pan troglodytes* pointing: Hand shapes, accuracy, and the role of eye gaze. J. Comp. Psych..

[42] Hopkins W.D., Leavens D.A. (1998). Hand use and gestural communication in chimpanzees Pan troglodytes. J. Comp. Psych..

[43] Savage-Rumbaugh E.S. (1986). Ape Language: From Conditioned Response to Symbol.

[44] Vèa J.J., Sabater-Pi J. (1998). Spontaneous pointing behaviour in the wild pygmy chimpanzee Pan paniscus. Folia Primatolog..

[45] Boysen S.T., Berntson G.G. (1989). Numerical competence in a chimpanzee, Pan troglodytes. J. Comp. Psych..

[46] Savage-Rumbaugh E.S., Susman R.L. (1984). Pan paniscus and Pan troglodytes: Contrast in preverbal communicative competence. The Pygmy Chimpanzee: Evolutionary Biology and Behavior.

[47] Patterson F.G., Peng F.C.C. (1978). Linguistic capabilities of a lowland gorilla. Sign Language and Language Acquisition in Man and Ape: New Dimensions in Comparative Pedolinguis tics.

[48] Miles H.L., Parker S.T., Gibson K.R. (1990). The cognitive foundations for reference in a signing orangutan. “Language” and Intelligence in Monkeys and Apes: Comparative Developmental Perspectives.

[49] Pepperberg I.M. (1999). The Alex Studies: Cognitive and Communicative Abilities of Grey Parrots.

[50] Pepperberg I.M. (2006). Grey parrot numerical competence: A review. Anim. Cogn..

[51] Reader S.M., Laland K.N. (2002). Social intelligence, innovation, and enhanced brain size in primates. PNAS.

[52] Maestripieri D., King B.J. (1999). Primate social organization, gestural repertoire size, and communication dynamics: A comparative study of macaques. The Evolution of Language: Assessing the Evidence from Nonhuman Primates.

[53] DeCasien A.R., Higham J.P. (2019). Primate mosaic brain evolution reflects selection on sensory and cognitive specialization. Nat. Ecol. Evol..

[54] Bard K.A., Maguire-Herring V., Tomonaga M., Matsuzawa T. (2019). The gesture ‘Touch’: Does meaning-making develop in chimpanzees’ use of a very flexible gesture?. Anim. Cogn..

[55] Locke D.P., Hillier L.W., Warren W.C., Worley K.C., Nazareth L.V., Muzny D.M. (2011). Comparative and demographic analysis of orang-utan genomes. Nature.

[56] Waterston R.H., Lindblad-Toh K., Birney E., Rogers J., Abril J.F., Agarwal P., Agarwala R., Ainscough R., Alexandersson M., An P. (2002). Initial sequencing and comparative analysis of the mouse genome. Nature.

[57] Kaplan G., Rogers L.J. (2004). Gene Worship. Moving beyond the Nature/Nurture Debate over Genes, Brain and Gender.

[58] Plumwood V., O’Reilly J., O’Reilly S., Sterling R. (1999). Being Prey. Ultimate Journey: Inspiring Stories of Living and Dying.

[59] Kaplan G. (2019). Holding up the mirror: Mirror neurons and humanity’s dark side. Anim. Sentience.

[60] Fouts R., Mills S.T. (1997). Next of Kin: What Chimpanzees Have Taught Me About Who We Are.

[61] Irvine L. (2004). A model of animal selfhood: Expanding interactionist possibilities. Symb. Interact..

[62] Hampton R. (2019). Parallel overinterpretation of behavior of apes and corvids. Learn. Behav..

[63] Hirata S., Fuwa K. (2007). Chimpanzees (Pan troglodytes) learn to act with other individuals in a cooperative task. Primates.

[64] Rohles F.H. (1961). The development of an instrumental skill sequence in the chimpanzee. J. Exp. Anal. Behav..

[65] Rohles F.H., Belleville R.E., Grunzke M.E. (1961). The measurement of higher intellectual functioning in the chimpanzee. Aerosp. Med..

[66] Cruickshank A.J., Gautier J.P., Chappuis C. (1993). Vocal mimicry in wild African grey parrots Psittacus erithacus. Ibis.

[67] Gautier J.P., Cruickshank A.J., Chappuis C. (1993). Vocal mimicry in wild African grey parrots Psittacus erithacus [sonograms recorded at Botsima, in the Salonga National Park, Zaire]. Annalen-Koninklijk Museum voor Midden-Afrika. Zool. Wet..

[68] Auersperg A.M., Borasinski S., Laumer I., Kacelnik A. (2016). Goffin’s cockatoos make the same tool type from different materials. Biol. Lett..

[69] Auersperg A.M.I., Köck C., O’Hara M., Huber L. (2018). Tool making cockatoos adjust the lengths but not the widths of their tools to function. PLoS ONE.

[70] O’Hara M., Mioduszewska B., Haryoko T., Prawiradilaga D.M., Huber L., Auersperg A. (2019). Extraction without tooling around—The first comprehensive description of the foraging-and socio-ecology of wild Goffin’s cockatoos (Cacatua goffiniana). Behaviour.

[71] Griffin D.R. (1978). Prospects for a cognitive ethology. Behav. Brain Sci..

[72] Marler P. (1996). Social Cognition. Curr. Ornithol..

[73] Ricklefs R.E. (2004). The cognitive face of avian life histories. Wilson Bull..

[74] Olkowicz S., Kocourek M., Lucan R.K., Portes M., Fitch W.T., Herculano-Houzel S., Nemec P. (2016). Birds have primate-like numbers of neurons in the forebrain. Proc. Natl. Acad. Sci. USA.

[75] Morimoto Y., Fujita K. (2011). Capuchin monkeys (Cebus apella) modify their own behaviors according to a conspecific’s emotional expressions. Primates.

[76] Kemp C., Kaplan G. (2013). Facial expressions in common marmosets (Callithrix jacchus) and their use by conspecifics. Anim. Cog..

[77] Caeiro C.C., Waller B.M., Zimmermann E., Burrows A.M., Davila-Ross M. (2012). Orang FACS: A muscle- based facial movement coding system for orangutans (Pongo spp.). Int. J. Primat..

[78] Vick S.J., Waller B.M., Parr L.A., Pasqualini M.C., Bard K.A. (2007). A cross-species comparison of facial morphology and movement in humans and chimpanzees using the facial action coding system (FACS). J. Nonverbal Behav..

[79] Julle-Daniere E., Micheletta J., Whitehouse J., Joly M., Gass C., Burrows A.M., Waller B.M. (2015). MaqFACS (Macaque Facial Action Coding System) can be used to document facial movements in Barbary macaques (Macaca sylvanus). PeerJ.

[80] Waller B.M., Lembeck M., Kuchenbuch P., Burrows A.M., Liebal K. (2012). GibbonFACS: A muscle-based facial movement coding system for hylobatids. Intern. J. Primatol..

[81] Waller B.M., Whitehouse J., Micheletta J. (2017). Rethinking primate facial expression: A predictive framework. Neurosc. Biobehav. Rev..

[82] Waller B.M., Micheletta J. (2013). Facial expression in nonhuman animals. Emot. Rev..

[83] Shettleworth S.J. (2003). Memory and hippocampal specialization in food-storing birds: Challenges for research on comparative cognition. Brain Behav. Evol..

[84] Clayton N.C., Vallortigara G., Emery N.J., Rogers L.J., Kaplan G. (2004). Comparative Vertebrate Cognition: Are Primates Superior to Non-Primates.

[85] Jarvis E.D., Güntürkün O., Bruce L., Csillag A., Karten H., Kuenzel W., Medina L., Paxinos G., Perkel D.J., Shimizu T. (2005). Avian brains and a new understanding of vertebrate brain evolution. Nat. Rev. Neurosc..

[86] Salwiczek L.H., Watanabe A., Clayton N.S. (2010). Ten years of research into avian models of episodic-like memory and its implications for developmental and comparative cognition. Behav. Brain Res..

[87] Homberger D.G., de Silva K.N. (2003). The role of mechanical forces on the patterning of the avian feather-bearing skin: A biomechanical analysis of the integumentary musculature in birds. J. Exp. Zool. Part B.

[88] Benitez-Quiroz C.F., Srinivasan R., Martinez A.M. (2018). Facial color is an efficient mechanism to visually transmit emotion. Proc. Natl. Acad. Sci. USA.

[89] Bertin A., Beraud A., Lansade L., Blache M.-C., Diot A., Mulot B., Arnould C. (2018). Facial display and blushing: Means of visual communication in blue- and-yellow macaws (Ara Ararauna)?. PLoS ONE.

[90] Kaplan G. (2007). Tawny Frogmouth.

[91] Dalziell A.H., Welbergen J.A., Igic B., Magrath R.D. (2015). Avian vocal mimicry: A unified conceptual framework. Biol. Rev..

[92] Kaplan G. (2017). Babbling in a bird shows same stages as in human infants: The importance of the ‘Social’ in vocal development. Trends Dev. Biol..

[93] Chakraborty M., Jarvis E.D. (2015). Brain evolution by brain pathway duplication. Philos. Trans. Roy Soc. B Biol. Sci..

[94] Güntürkün O., Bugnyar T. (2016). Cognition without cortex. Trends Cogn. Sci..

[95] Kaplan G. (2019). Bird Bonds. Sex, Mate-Choice and Cognition. Australian Native Birds.

[96] Kleiner K. (2002). Review: Steven Wise’s “Drawing the Line”: Science and the Cases for Animal Rights.

[97] Plumwood V. (2007). Human exceptionalism and the limitations of animals: A review of Raimond Gaita’s “The philosopher’s dog”. Aust. Humanit. Rev..

[98] Gaita R. (2002). The Philosopher’s Dog.

[99] Bekoff M. (2000). Animal emotions: Exploring passionate natures. Bioscience.

[100] Birch J. (2020). Review Essay: The place of animals in Kantian ethics Christine, M.Korsgaard. Fellow creatures: Our obligations to other animals. Biol. Philos..

